# Omega-3 index and smoking in patients with acute ST-elevation myocardial infarction taking statins: a case-control study in Korea

**DOI:** 10.1186/1476-511X-11-43

**Published:** 2012-03-27

**Authors:** Young Joo Kim, Dong Wook Jeong, Jeong Gyu Lee, Han Cheol Lee, Sang Yeoup Lee, Yun Jin Kim, Yu Hyeon Yi, Yong Soon Park, Young Hye Cho, Mi Jin Bae, Eun Jung Choi

**Affiliations:** 1 Department of Family Medicine, Pusan National University Hospital, Busan, South Korea; 2 Family Medicine Clinic and Research Institute of Convergence of Biomedical Science and Technology, Pusan National University Yangsan Hospital, Yangsan, South Korea; 3 Division of Cardiology, Department of Internal Medicine, Pusan National University Hospital, Busan, South Korea; 4 Medical Education Unit and Medical Research Institute, Pusan National University School of Medicine, Yangsan, South Korea; 5 Department of Food & Nutrition, Hanyang University, Seoul, South Korea; 6 Family Medicine Clinic, Pusan National University Yangsan Hospital, Beomeo-ri, Mulgeum-eup, Yangsan, Gyeongsangnam-do 626-770, South Korea

**Keywords:** Omega-3 index, Smoking, Myocardial infarction, Fatty acid, Fish oil

## Abstract

**Background:**

n-3 fatty acids and lifestyle also are closely related to risk of CVD. Most Koreans have higher fish consumption than people of Western populations. However, little is known about the recommended value of omega-3 index in Korean patients with acute ST-elevation myocardial infarction (STEMI) taking statins. Here, we tested the hypothesis that lower omega-3 fatty acids and/or smoking are associated with acute STEMI, even though patients with dyslipidemia who were taking statins and who attained their LDL-C goals.

**Methods:**

We conducted a case-control study in which omega-3 fatty acids and lifestyle factors were determined in 24 consecutive Korean patients taking statins with angiographically confirmed acute STEMI and 68 healthy controls without acute STEMI. The omega-3 index was calculated by the sum of eicosapentaenoic acid and docosahexaenoic acid in erythrocyte membranes. Multivariable adjusted regression analysis was used to assess independent associations between acute STEMI, omega-3 index, and lifestyle factors after adjusting for age, sex, and body mass index (BMI).

**Results:**

The mean age of total subjects was 59.9 years, and 57.6% of the subjects were male. The omega-3 index was significantly lower in cases (8.83%) than controls (11.13%; P < 0.001); however, total *trans*-fatty acids were not different between the two groups. The omega-3 index was inversely associated with odds for being a case (OR 0.16 (95% CI 0.03-1.14); P = 0.047), while smoking was positively associated with odds for being a case (OR 6.67 (95% CI 1.77-25.23); P = 0.005) after adjusting for all confounding variables.

**Conclusion:**

This study shows that relative to controls, acute STEMI cases are more likely to be smokers and to have a lower omega-3 index, even though the cases were taking statins. An omega-3 index of at least 11% and abstinence from smoking are associated with cardioprotection for Koreans.

## Background

Cardiovascular disease (CVD) is one of the most common causes of mortality and morbidity across the globe [1]. Studies that have focused on the prevention of coronary heart disease (CHD) have reported that lifestyle modifications such as smoking cessation, adequate alcohol consumption, exercise, stress reduction, weight control, and diet are preventable factors of CVD [2-5]. Dietary *n-3 *polyunsaturated fatty acids (PUFA) such as eicosapentaenoic acid (EPA) and docosahexaenoic acid (DHA), which are found in fish meat and fish oil, have also been shown to protect against CVD during the past three decades [6]. Since the first observation of Greenland Eskimos in 1970, epidemiologic studies have suggested that *n-3 *PUFA may play a role in the prevention of CVD [7]. Several large-scale intervention studies have reported that *n-3 *PUFA from fish or fish-oil supplements reduces the rates of all-cause mortality, cardiac and sudden death, and possibly stroke [8]. In addition, *n-3 *fatty acids are considered to have several beneficial effects, including reduction of inflammation [9], prevention of blood clotting [10], decrease in triglycerides [11], and lowering of blood pressure [12].

The sum of EPA + DHA in erythrocyte (RBC) membrane fatty acids is a good reflection of systemic n-3 PUFA status and is called the omega-3 index, which is expressed as a percent of total identified fatty acids [13]. Previous studies have shown that Asians with higher fish consumption had a greater omega-3 index, and furthermore, they had a higher omega-3 index than the recommended value of 8% or greater to prevent CHD for Western people. The omega-3 index in healthy Koreans and Japanese is about 11-12 and 7-11%, respectively, while it is around 4% in people from Western populations [13-15]. Nevertheless, CVD is becoming more prevalent in Korea and is now a leading cause of death [16], although the prevalence of CVD in Korea remains lower than that in the US and other Western populations. A study of more than 40,000 middle-aged Japanese people revealed that a higher fish intake is associated with a substantially reduced risk of CHD compared to the recommended amount of fish intake in Western countries [17].

A recent meta-analysis of 3-hydroxy-3-methylglutaryl coenzyme A reductase inhibitors (statins) in primary prevention showed that the lipid profile was improved in all 14 trials of about 34,000 people who had no evidence of existing CVD, but the benefits of statins for the primary prevention of CVD may differ according to cardiovascular risk [18]. Recently, several studies confirmed that prescription omega-3 fatty acids administered in statin-treated patients with hypertriglyceridemia was superior to statins alone in improving lipid parameters [19-21]. However, it remains unclear whether omega-3 index is different between controls and patients with recent myocardial infarction (MI) taking statins in Koreans, who consume more fish than Western populations. The purpose of the present study was to compare fatty acid composition of RBCs, the omega-3 index, and lifestyle behaviors between controls and patients with acute ST-elevation MI (STEMI) who attained their LDL-C goals with statins.

## Materials and methods

### Study design and subjects

This was a cross-sectional, case-control study. The study protocol was approved by the Institutional Review Board of Pusan National University Hospital, and informed written consent was obtained from all subjects before participating.

Subjects were divided into 2 subgroups: a case group of 24 patients with first acute MI, who were consecutively recruited from the Coronary Care Unit at Pusan National University Hospital (Busan, Korea) and a control group of 68 apparently healthy persons with no history or clinical evidence of CVD, hypertension, or diabetes mellitus as well as acute MI, who were not taking statins and recruited from the Health Promotion Center during the course of annual health check-up visits.

Emergency coronary angiography was performed using the percutaneous femoral approach within the first 12 h after the onset of typical ischaemic-type chest pain. Acute STEMI was defined according to consensus documents of American College of Cardiology and European Society of Cardiology as typical ischaemic-type chest pain, typical rise and fall of biochemical markers (troponin or CK-MB), and electrocardiogram showed new ST segment elevation or pathologic Q waves. The number and severity of coronary artery stenosis were documented through coronary angiography. All patients were already taking statins due to dyslipidemia with/without hypertension or type 2 diabetes mellitus. Exclusion criteria included abnormal liver or renal function (ie, serum aminotransferase activity > 40 IU/L and serum creatinine concentrations > 1.2 mg/dL); cancer (diagnosed clinically or by anamnesis); extreme weight loss or gain over the previous 6 months; thyroid or pituitary disease; infection determined by medical questionnaire and complete blood count; and connective tissue disease.

Clinical, sociodemographic, lifestyle, and medication information such as oral hypoglycemic agents, insulin, lipid-lowering agents, and anti-hypertensive or estrogen agents were obtained from all study subjects. Subjects taking a supplement containing n-3 fatty acids were excluded.

### Measurements

Height and body weight of subjects were measured using a digital scale, and the body mass index (BMI) was calculated as weight (kg)/height (m^2^). Resting blood pressure was measured using an automatic sphygmomanometer (BP203RV-II; Nippon Colin, Komaki, Japan) after > 10 min at rest in a sitting position.

Peripheral blood was drawn from patients on day 1 (at the time of admission) after the onset of infarction and controls after an overnight fast of at least 8 h for measurements of serum total cholesterol, triglycerides, high density lipoprotein (HDL)-cholesterol, low density lipoprotein (LDL)-cholesterol, and glucose. All biochemical analyses were carried out within 2 hr of blood sampling using an autoanalyzer (model 7600-110; Hitachi Corp., Tokyo, Japan) and commercially available kits. RBCs were used for fatty acid analysis. Boron trifluoride methanol-benzene (BI252; Sigma-Aldrich, BI252; Sigma-Aldrich, St. Louis, MO, USA) was added to RBCs, and samples were methylated for 10 min at 100°C. Fatty acid methyl esters were analyzed by gas chromatography (Shimadzu 2010 AF; Shimadzu Scientific Instrument, Tokyo, Japan) with a 100 mm × 0.25 mm SP2560 capillary column (Supelco; Bellefonte, PA, USA). Standard gas liquid chromatography (GLC-727; Nu-Check Prep, Elysian, MN, USA) was used for identifying fatty acids and correcting inter-assay variation. In the standard 18: 1t peak was the mixture of 18: 1*n*-12t, C18: 1*n*-9t, and 18: 1*n*-7t, while the 18: 2*n*-6t peak contained 18: 2*n*-6tt, 18:2n-6tc, and 18:2n-6ct. Fatty acids for which no standards were available are reported as unidentified fatty acids. When the percentage of fatty acid was less than 0 5, it is recorded as a trace. The omega-3 index was calculated as the sum of EPA and DHA in RBCs and expressed as percentage of total fatty acids in the RBC membrane [22]. The quality-control sample was composed of pooled RBCs, and the coefficient of variation was 6.2%.

The usual dietary intakes were assessed using a semi-quantitative food frequency questionnaire (FFQ). A trained dietitian interviewed the participants regarding the average frequency of consumption and portion size of each food weekly. FFQ was designed to assess the habitual diet during the previous seven days, the daily intakes of food groups, energy, and nutrients were computed using an analysis program, and weekly total fish intakes were obtained from all subjects. Alcohol consumption and smoking status were assessed using a self-reported questionnaire. Data on alcohol intake and smoking habits were obtained by interview. Subjects were divided into two groups by the amount of alcohol consumption: non-drinker, 0-180 g/week; and drinker, > 180 g/week. Based on WHO guidelines, smoking status was divided into three categories: current smoker, ex-smoker, and non-smoker.

### Statistical analysis

The D'Agostino-Pearson test was used to test the normality of all continuous variables. Results are shown as mean and s.d. because variables were all normally distributed. The two sample *t*-test and chi-square test were applied for continuous and categorical variables, respectively. Correlation between variables was tested by partial correlation coefficients after adjusting for age and sex. Multivariable adjusted regression analysis was used to assess independent associations between acute STEMI, quartiles of the omega-3 index, and lifestyle factors after adjusting for age, sex, and BMI. Statistical analysis was obtained using the Statistical Package for Social Science 13.0 for Windows (SPSS, Inc., Chicago, USA). All p values were two-tailed, and a p value of < 0.05 was considered statistically significant.

## Results

### General characteristics of study subjects

The basal characteristics of subjects are shown in Table 1. Among all acute STEMI patients having multi-vessel coronary artery disease, 12 (50.0%) had one-vessel disease, 5 (20.8%) had two-vessel disease, and 7 (29.2%) had three-vessel disease. The mean age of total subjects was 59.9 ± 9.1 years, and 57.6% of the subjects were male. Looking at the case-control comparison, the case group had significantly lower levels of total cholesterol, LDL-cholesterol, HDL-cholesterol, and systolic blood pressure and higher levels of high sensitivity C-reactive protein than did the control group. However, there was no significant difference in age, gender distribution, BMI, triglyceride, and diastolic blood pressure between the two groups. There was a significantly higher percentage of current smokers in the case group than in the control group, while there was no difference in the proportion of alcohol drinkers between the two groups. All cases had dyslipidemia with/without hypertension or diabetes mellitus and received statins.

**Table 1:** Sociodemographic and clinical characteristics of study subjects

	Case (n = 24)	Control (n = 68)	p value
Age (years)	61.6 ± 11.6	59.3 ± 8.1	0.370
Men (%)	17 (70.8)	36 (52.9)	0.127
Body mass index (kg/m^2^)	23.5 ± 2.8	24.0 ± 2.5	0.405
Total cholesterol (mg/dl)	159.0 ± 30.0	201.1 ± 38.8	< 0.001
Triglyceride (mg/dl)	123.3 ± 73.9	128.7 ± 62.8	0.731
HDL-cholesterol (mg/dl)	42.1 ± 10.8	52.9 ± 10.7	< 0.001
LDL-cholesterol (mg/dl)	81.4 ± 24.7	119.8 ± 34.7	< 0.001
Systolic BP (mm/Hg)	111.9 ± 18.7	124.3 ± 14.1	0.001
Diastolic BP (mm/Hg)	70.5 ± 13.0	76.0 ± 8.6	0.065
hs-CRP (mg/dl)	2.66 ± 3.50	0.13 ± 0.19	0.002
Co-morbidity			
Hypertension	10 (41.7)	0 (0.0)	< 0.001
Diabetes mellitus	12 (50.0)	0 (0.0)	< 0.001
Dyslipidemia	24 (100.0)	0 (0.0)	< 0.001
Fish intake (servings/week)	2.1 ± 2.1	3.1 ± 2.1	0.059
Alcohol drinker (%)	10 (41.7)	21 (30.9)	0.337
Current smoker (%)	11 (45.8)	10 (14.7)	0.002

Data are expressed as means ± SD or number (%).

HDL, high density lipoprotein; LDL, low density lipoprotein; BP, blood pressure; hs-CRP, high sensitivity C-reactive protein.

p value by two sample *t*-test or chi-square test

### RBC membrane fatty acid composition

Fatty acid composition of RBCs is presented in Table 2. No difference was observed regarding the RBC membrane contents of saturated fatty acids, PUFA, and total *trans*-fatty acids between the case-control groups, but monounsaturated fatty acids (MUFA) of RBC were significantly lower in cases than controls (P = 0.011). Among saturated fatty acids, 18: 0 was lower in cases when compared to that found in controls and 24: 0 was higher. In regard to MUFA, 16: 1n-7t, 18: 1n-9, and 20: 1n-9 were significantly higher in cases than controls, whereas 16: 1n-7 was lower. Of RBC contents of PUFA, omega 6 fatty acids were significantly higher in cases than in controls (P < 0.001), while omega 3 fatty acids were lower (P = 0.008). In omega 6 fatty acids, only 18: 2n-6t was lower in cases than in controls, but 18: 2n-6 and 20: 3n-6 were higher. Cases had significantly lower RBC membrane contents of 20: 5n-3 (EPA), 22: 5n-3, and 22: 6n-3 (DHA) than did controls. The omega 6/omega 3 ratio and arachidonic acid/EPA ratio were significantly higher, but the omega-3 index was significantly lower in cases than in controls (P = 0.002).

**Table 2:** Fatty acid composition of erythrocytes in subjects

Fatty acids (weight %)	Case (n = 24)	Control (n = 68)	p value*
Saturated fatty acids	39.45 ± 6.45	40.12 ± 4.90	0.600
Myristic (14:0)	0.77 ± 0.41	0.65 ± 0.43	0.227
Palmitic (16:0)	24.31 ± 3.44	23.63 ± 3.30	0.390
Stearic (18:0)	13.71 ± 3.64	15.34 ± 2.79	0.025
Lignoceric (24:0)	0.67 ± 0.47	0.43 ± 0.51	0.048
Monounsaturated fatty acids	17.09 ± 1.91	18.22 ± 1.81	0.011
*trans*-Palmitoleic (16:1n-7t)	0.60 ± 0.55	0.36 ± 0.42	0.031
Palmitoleic (16:1n-7)	1.11 ± 0.48	4.40 ± 2.87	< 0.001
*trans*-Oleic (18:1n-9t)	0.84 ± 0.41	0.58 ± 0.65	0.071
Oleic (18:1n-9)	14.65 ± 1.70	12.96 ± 2.43	0.002
Eicosenoic (20:1n-9)	0.79 ± 0.50	0.31 ± 0.37	< 0.001
Nervonic (24:1n-9)	0.54 ± 0.45	0.36 ± 0.88	0.062
Polyunsaturated fatty acids	41.35 ± 7.36	40.01 ± 5.45	0.351
Omega-6	29.81 ± 5.01	26.05 ± 4.14	< 0.001
*trans*-Linoleic (18:2n-6t)	0.22 ± 0.17	0.57 ± 0.51	< 0.001
Linoleic (18:2n-6)	14.38 ± 4.59	10.54 ± 3.42	< 0.001
Gamma-linolenic (18:3n-6)	0.34 ± 0.18	0.27 ± 0.56	0.590
Eicosadienoic (20:2n-6)	0.33 ± 0.18	0.41 ± 0.48	0.425
Dihomo-GLA (20:3n-6)	1.72 ± 0.46	1.43 ± 0.38	0.003
Arachidonic (20:4n-6)	11.25 ± 3.07	11.25 ± 2.76	0.998
Adrenic (22:4n-6)	1.29 ± 0.60	1.53 ± 0.61	0.101
Docosapentaenoic (22:5n-6)	0.51 ± 0.30	0.48 ± 0.61	0.753
Omega-3	11.54 ± 3.58	13.96 ± 3.86	0.008
Alpha-linolenic (18:3n-3)	0.77 ± 0.43	0.60 ± 0.66	0.148
Eicosapentaenoic (20:5n-3)	1.80 ± 0.65	2.53 ± 0.96	< 0.001
Docosapentaenoic (22:5n-3)	1.93 ± 0.73	2.64 ± 0.91	0.001
Docosahexaenoic (22:6n-3)	7.03 ± 2.54	8.61 ± 2.57	0.011
n-6/n-3	2.79 ± 0.83	2.07 ± 0.86	0.001
Omega-3 index	8.83 ± 3.00	11.13 ± 3.08	0.002
AA/EPA	6.88 ± 2.84	5.07 ± 2.20	0.002
Total *trans *fatty acids	1.51 ± 0.68	1.39 ± 0.86	0.527

*By two sample *t*-test; Omega-3 index, docosahexaenoic acid + eicosapentaenoic acid %; AA, arachidonic acid; EPA, eicosapentaenoic acid

### Correlation between acute STEMI, alcohol drinking, smoking, BMI, and RBC membrane fatty acid composition

The partial correlation coefficients between acute STEMI, alcohol drinking, smoking, BMI, and RBC membrane fatty acid composition are shown in Table 3. There was a negative correlation between acute STEMI and quartiles of the omega-3 index (r = -0.585, P < 0.001), BMI (r = -0.272, *P *= 0.027), and MUFA (r = -0.299, P = 0.015), while there was a borderline correlation between acute STEMI and fish consumption (r = -0.242, P = 0.050) after adjusting for age and gender. However, acute STEMI correlated positively with smoking (r = 0.366, P = 0.003) and the arachidonic acid/EPA ratio (r = 0.309, *P *= 0.012) after adjusting for age and gender.

**Table 3:** Correlation between acute myocardial infarction, alcohol drinking, smoking, body mass index, and erythrocyte membrane fatty acid composition

Variables	Acute MI	Omega-3 index
Omega-3 index	**-0.585 (< 0.001)**	
Alcohol drinking	-0.004 (0.973)	-0.121 (0.333)
Smoking	**0.366 (0.003)**	-0.220 (0.076)
Body mass index	**-0.272 (0.027)**	**0.293 (0.017)**
Fish intake (servings/week)	**-0.242 (0.050)**	**0.284 (0.021)**
Total saturated fatty acids	-0.049 (0.693)	**-0.424 (< 0.001)**
Total monounsaturated fatty acids	**-0.299 (0.015)**	-0.213 (0.086)
Total polyunsaturated fatty acids	0.080 (0.503)	**0.499 (< 0.001)**
Arachidonic acid/eicosapentaenoic acid ratio	**0.309 (0.012)**	**-0.473 (< 0.001)**
Total *trans *fatty acids	0.117 (0.350)	-0.150 (0.231)

By partial correlation coefficient after adjustment for age and gender

Data are expressed as correlation coefficients (p value).

### Association of the omega-3 index, smoking, and odds for being acute STEMI

Study subjects were categorized into four groups according to the omega-3 index. Multivariable adjusted regression analysis showed that the omega-3 index, age; the omega-3 index was inversely associated with odds for being a case (OR 0.16 (95% CI 0.03-1.14); P = 0.047), while smoking was positively associated with odds for being a case (OR 6.67 (95% CI 1.77-25.23); P = 0.005) after adjusting for age, gender, BMI, alcohol drinking (Table 4).

**Table 4:** Multivariate analysis of the odds for being a case by quartiles of the Omega-3 Index

	Acute myocardial infarction	
Variables	Odds ratio (p value)	**95% C.I**.
Omega-3 index (%)		
1^st ^quartile (< 8.11)*	6.383 (0.047)	1.022 - 39.853
2^nd ^quartile (8.11-11.00)	2.032 (0.471)	0.072 - 14.001
3^rd ^quartile (11.00-13.05)	2.982 (0.258)	0.105 - 19.823
4^th ^quartile (> 13.05)	1.000	
Age*	1.073 (0.032)	1.006 - 1.144
Sex	1.301 (0.701)	0.340 - 4.980
Body mass index	1.019 (0.866)	0.822 - 1.262
Alcohol drinker	1.466 (0.568)	0.378 - 5.894
Current Smoker*	6.673 (0.005)	1.765 - 25.229

*p < 0.05, Test for trend across quartiles of Omega-3 index (P = 0.01).

## Discussion

Statins are well-known as the first-line drugs in the treatment of hyper-LDL cholesterolemia. Large-scale, prospective, randomized clinical trials have demonstrated that statins improve clinical cardiovascular outcomes and reduce mortality in both primary and secondary prevention [18]. However, in the real world, despite that clinical evidence, CHD including acute MI can occur even among dyslipidemia patients currently taking a statin with an LDL goal of less than 100 mg/d [23]. Therefore, combining omega-3 fatty acids with a statin induces further triglyceride reduction for patients with high triglyceride levels despite achieving target LDL cholesterol goals. However, little is known about further prevention of cardiovascular events in hypercholesterolemia patients without hypertriglyceridemia, especially with the addition of fish oil capsules to statin therapy. A study did report, however, that there was a further decrease in nonfatal coronary events or unstable angina in patients with hypercholesterolemia, especially in secondary prevention with the use of statins and EPA in combination therapy [24]. This was the first study to show an additional clinical benefit of long-term combination therapy with omega-3 fatty acids and statins without serious adverse effects compared to statins only. However, this trial was not designed as a double-blind study. Also, unfortunately, the study did not show RBC fatty acid composition, investigated only EPA and not a DHA effect on coronary events, and revealed no additional benefit for acute MI.

Omega-3 fatty acids are known to cause improvement in lipid profiles without inducing serious hepatotoxicity or myopathy. Unlike previous studies, our study was specifically designed to evaluate the fatty acid composition of RBC and lifestyle factors such as alcohol consumption and smoking status in patients who developed acute STEMI, although they were taking statins and had achieved their LDL cholesterol control. The findings of this study can give us better understanding of the effectiveness and limitation of statin use for primary prevention of acute MI.

In the present study, the average omega-3 index in healthy controls was about 11%, which was similar to figures from previous studies in Koreans [14,25], and slightly higher than those reported by previous studies performed in Japan [26,27]. Recent studies have shown that the Japanese have an omega-3 index of 7-11%, and the index tends to rise along with increasing age [26,27]. Our study did not show a correlation between omega-3 index and age.

Recently, two case-control studies were conducted to reveal the omega-3 index of RBCs in patients with acute MI or metabolic syndrome in Korea. One study found that patients with acute MI have a lower omega-3 index (9.6%) in comparison to healthy controls, and total *trans*-fatty acids are associated with an increased risk of MI. However, the result did not have adequate statistical power due to a wide confidence interval of the odds ratio of MI risk estimate. The other study reported that patients with (11.8%) and without metabolic syndrome (12.4%) have similar omega-3 indices. In the present study, we found that patients who experienced acute STEMI, even though they were taking statins and had achieved their LDL cholesterol control goal, had an omega-3 index of 8.8%, which was significantly lower than that of controls (11.1%), while no difference in total *trans*-fatty acids was observed between the two groups. Previous studies have provided inconsistent data on whether *trans *fat intakes are associated with MI risk according to levels of intake [28-30]. On the other hand, the present study found that omega-6 fatty acids such as linoleic acid were increased in patients with acute STEMI compared to controls, which was in accordance with a previous study [31]. Linoleic acid was frequently inversely associated with risk for coronary heart disease events in Western people [32]. However, the present study found that omega-6 fatty acids such as linoleic acid were increased in patients with acute STEMI compared to controls. Previous studies from Korea and Japan showed that there were no differences of linoleic acid level between MI cases and controls [25,33]. Although it is not yet known why, possible mechanisms underlying the ethnic difference in linoleic acid concentrations include differences in fat distribution, fatty acid interactions with genetic polymorphisms, and diet. Replication studies with larger sample size would be needed to confirm our study results.

The American Heart Association recommends that healthy people should eat two servings of a variety of (preferably oily) fish per week [11]. The present study showed an association between fish consumption and the omega-3 index (r = 0.269, P = 0.026), although two sample *t*-test revealed only a borderline difference of average fish consumption (servings/week) between cases and controls (2.1 vs. 3.1, P = 0.059), although the consumption was greater than that in most Western populations. A Japanese study also showed that a very high level of fish intake was significantly associated with a lower risk of nonfatal CHD [17].

Harris et al. [13] suggest the omega-3 index (EPA + DHA as a percent of total fatty acid in RBC membranes) as a novel risk factor for CHD. The studies have shown that a higher omega-3 index than a value of 8-10% is desirable for its cardio-protective benefits, because of the relationship between a lower average omega-3 index and the development of CHD in Caucasians [13,22]. Interestingly, Korean patients with acute STEMI already had an omega-3 index of 8%, which is a cardio-protective goal for Western people. Therefore, we suggest that a higher cut-off point of omega-3 index than 8-10% for preventing CHD is needed, and an omega-3 index of 11% or above could be appropriate in Korea. This discrepancy between Korean and Western people could be explained in large part by the fact that most Korean people have higher fish consumption than people of Western populations. Korean consumes fish species such as mackerel, salmon which are very high in omega-3 [34]. In addition, there may be ethnic differences in the threshold of omega-3 index and underlying mechanism of development of CHD. Using multivariable adjusted regression analysis, our study showed that lower omega-3 index was independently associated with acute STEMI after adjusting for age, sex, and BMI. Subjects in the lowest quartile of omega-3 index was positively associated with almost six times odds for being a case, even after adjusting for all confounding variables, than the highest quartile subgroup (Table 4). That finding is concordant with previous case-control studies about EPA and DHA levels that found significantly lower EPA and DHA in patients with acute coronary syndrome compared to healthy control people [29,35]. A case-control follow-up study also provides evidence of a plasma concentration of EPA and DPA associated with a lower incidence of nonfatal MI among American women [30], despite their current lower fish intake than that of typical Korean and Japanese people.

Cigarette smoking is an important risk factor for cerebro-cardiovascular diseases. Epidemiologic, clinical, and experimental data have revealed that smoking is prothrombotic, atherogenic, and causative factors in the development of CHD [36]. In the present study, we also found that smoking was a major associated factor for acute STEMI development in patients taking statins who had achieved their goal LDL cholesterol. Our results were consistent with a previous study using Japanese data that revealed an association of cigarette smoking with CVD mortality in patients, despite their lower total cholesterol level [37]. The relationship between omega-3 index and smoking status is a controversial topic [38]. In additional analysis, we found no difference in the omega-3 index between smokers and non-smokers within each group (data not shown).

There is no single cause for CHD. CVD is caused by a constellation of risk factors, including environmental factors, such as sedentary lifestyle, genetic predisposition, advancing age, smoking, an atherogenic diet, and underlying conditions or diseases including insulin resistance or type 2 diabetes, hypertension, and dyslipidemia. In this view, interestingly, the present study showed that smoking, a lower omega-3 index, and advancing age together among those factors may work together to cause acute STEMI despite statin-induced low LDL cholesterolemia. Another Korean study also reported that, compared to survivors, non-survivors of acute MI had a lower level of total cholesterol, triglycerides, and LDL-cholesterol, while the opposite was observed with plasma omega-3 fatty acids [39]. However, the researchers did not explain why non-survivors from acute MI had lower lipid levels than survivors. It is obvious, however, that certain cardiovascular events still occur even among patients reaching target levels of LDL-cholesterol [30,35]. Therefore, relative to controls, STEMI cases are more likely to be smokers and to have a lower omega-3 index, even though the LDL cholesterol target is achieved with a statin in the cases with dyslipidemia. Patients should be asked to quit smoking and eat more fish or take omega-3 supplements as well as reach their LDL-cholesterol target on a statin.

Our study is limited by its cross-sectional study design. Further investigation with a cohort study is warranted. Another limitation is the wide confidence interval due to a relatively small sample size. Koreans usually have a fish intake that is several times higher than Western people, which limits the generalizability of those results to other population groups.

In summary, subjects with acute STEMI had a lower omega-3 index and higher smoking rate after adjusting for age, gender, and BMI than controls, although they are currently receiving a statin with an LDL goal of less than 100 mg/d and normal triglyceride levels. Nevertheless, acute STEMI patients have an omega-3 index of 8.8% that is higher than that of people of Western populations. This means that the cut-off point for the omega-3 index for preventing CVD might be tailored for the Korean population. Thus, at present, we suggest a suitable level of an omega-3 index of 11% or above for prevention of coronary artery disease in the Korean population. However, replication studies with a larger sample size are needed to confirm our first stage study results for current practice in the field.

## Competing interests

All authors have completed and submitted the ICMJE Form for Disclosure of Potential Conflicts of Interest.

## Authors' contributions

SYL had full access to all of the data in the study and takes responsibility for the integrity of the data and the accuracy of the data analysis. YJK, JGL and SYL participated in the study concept and design. YJK, HCL and YHC participated in acquisition of data. YJK, SYL and YSP participated in analysis and interpretation of data. SYL and JGL participated in drafting of the manuscript. SJI, MJB and EJC participated in critical revision of the manuscript for important intellectual content. SYL and DWJ carried out statistical analysis. JGL, YHC, MJB and EJC participated in administrative, technical, or material support. All authors read and approved the final manuscript.

## Funding/Support

This work was supported by a Medical Research Institute Grant (2007-9) from Pusan National University, Busan, Korea
